# Electrospun ZnO/Pd Nanofibers: CO Sensing and Humidity Effect

**DOI:** 10.3390/s20247333

**Published:** 2020-12-20

**Authors:** Vadim Platonov, Marina Rumyantseva, Nikolay Khmelevsky, Alexander Gaskov

**Affiliations:** 1 Chemistry Department, Moscow State University, 119991 Moscow, Russia; agnes1992@yandex.ru (V.P.); gaskov@inorg.chem.msu.ru (A.G.); 2 Institute of Chemistry, Saint Petersburg State University, Petergof, 198504 Saint Petersburg, Russia; 3 Material Properties Research Laboratory (LISM), Moscow State Technological University Stankin, 127055 Moscow, Russia; khmelevsky@mail.ru

**Keywords:** zinc oxide, Pd clusters, electrospinning, nanofibers, semiconductor gas sensor, carbon monoxide, humidity

## Abstract

Variable air humidity affects the characteristics of semiconductor metal oxides, which complicates the reliable and reproducible determination of CO content in ambient air by resistive gas sensors. In this work, we determined the sensor properties of electrospun ZnO and ZnO/Pd nanofibers in the detection of CO in dry and humid air, and investigated the sensing mechanism. The microstructure of the samples, palladium content, and oxidation state, type, and concentration of surface groups were characterized using complementary techniques: X-ray fluorescent spectroscopy, XRD, high-resolution transmission electron microscopy (HRTEM), high angle annular dark field scanning transmission electron microscopy (HAADF-STEM), energy-dispersive X-ray (EDX) mapping, XPS, and FTIR spectroscopy. The sensor properties of ZnO and ZnO/Pd nanofibers were studied at 100–450 °C in the concentration range of 5–15 ppm CO in dry (RH_25_ = 0%) and humid (RH_25_ = 60%) air. It was found that under humid conditions, ZnO completely loses its sensitivity to CO, while ZnO/Pd retains a high sensor response. On the basis of in situ diffuse reflectance IR Fourier transform spectroscopy (DRIFTS) results, it was concluded that high sensor response of ZnO/Pd nanofibers in dry and humid air was due to the electronic sensitization effect, which was not influenced by humidity change.

## 1. Introduction

The need for CO quantification for early fire detection, monitoring the completeness of fuel combustion, and exhaled breath analysis in medical diagnostics is constantly increasing. The influence of variable air humidity on the characteristics of semiconductor metal oxides is one of the important negative factors which complicates the reliable and reproducible determination of CO in ambient air by resistive gas sensors. Since water vapor interacts with the surface of n-type semiconductor oxides in a similar way to reducing gases, i.e., causing a decrease of the resistance of the material, it significantly complicates the detection of target gases [1,2,3]. The negative effects of H_2_O include the deteriorated selectivity and sensitivity, and longer response and recovery times. Changes in ambient humidity during long-term operation cause deviations of the actual parameters of semiconductor gas sensors from the calibration characteristics. Currently, the most efficient way to improve the accuracy, reproducibility, and reliability of the results of air analysis by semiconductor gas sensors is the development of multi-sensor arrays containing several sensing elements with the use of an appropriate dynamic temperature regime and various mathematical algorithms for processing the sensor signal [4,5,6,7]. Tuning the properties of the sensitive material toward various groups of gases allows one to further improve the sensor properties of the system when solving specific analytical tasks. An important challenge is the minimization of the materials sensitivity to humidity changes.

In the case of ZnO, using the methods of He atom scattering (HAS) and IR spectroscopy [8] it was found that water was chemisorbed as monolayer and bilayer adsorbed H_2_O molecules on the single-crystal polar surface of O–ZnO. The formation of surface OH groups is possible on the non-polar surface of zinc oxide, for example, ZnO ($10\bar{1}0$). Studies of water adsorption using high-resolution electron energy loss spectroscopy (HREELS), HAS, and thermal desorption methods have shown that some water molecules occur directly in the form of H_2_O, while others are in the dissociated form [9]. According to IR spectroscopy, the interaction of water vapor with nanocrystalline ZnO at 50 °C is realized through both molecular and dissociative chemisorption of H_2_O [10]. The following processes were identified: (i) the formation of OH groups on the polar surface of O-ZnO when water dissociates on oxygen vacancies; (ii) partial dissociation of water on the surface of ZnO ($10\bar{1}0$) with the formation of chemisorbed H_2_O molecules and OH groups; (iii) the appearance of isolated OH groups on the surface of ZnO ($10\bar{1}0$); (iv) the interaction of water with defects with the formation of O-H...O bonds [10].

The authors [11] investigated the sensor properties of one-dimensional ZnO nanoneedles towards CO in various gas concentrations and relative humidity. With an increase of humidity when CO is detected at room temperature the sensor response increased, but longer response and relaxation times were noted. In the work [12], the characteristics of gas sensors based on zinc oxide with rod- and needle-like morphology were compared. It was found that when detecting CO and H_2_ at 400 °C, the needle-like systems showed lower sensitivity to humidity and higher sensor signals to the analyzed gases. These results were explained by the differences in the chemical composition of the surface of rod- and needle-like zinc oxide. A higher concentration of oxygen vacancies and a higher surface-to-volume ratio were assumed for the needles than for the rods. The gas sensor properties of ZnO thin films synthesized by spray chemical vapor deposition (spray-CVD) technique were studied when detecting NO_2_, CO, and NO at 400–500 °C in dry and humid air [13]. Gas sensor tests in dry air showed high sensor response to NO_2_ and CO and low response to NO. The maximum sensor signals were observed at the measurement temperature of 450 °C. The increasing humidity from RH = 2.5% to RH = 10% lead to a slight decrease of the sensor response to NO_2_ and a dramatic decrease of the sensitivity to CO. The authors attributed this difference to the presence of stronger van der Waals interactions between hydroxyl groups and NO_2_, compared to the interactions with CO.

The sensor properties of semiconductor oxides can be enhanced by functionalizing their surface with noble metal nanoparticles (for example, Pd, Pt, and Au). A “chemical mechanism” or “electronic mechanism” is usually used to describe the improved sensor response of catalytically functionalized metal oxides. The chemical mechanism involves oxygen spillover and/or specific oxidation of the target gas with participation of noble metal nanoparticles. This effect reduces the operating temperature of semiconductor gas sensors, as well as increases the sensitivity and selectivity. When the electronic mechanism is implemented, a Schottky junction is formed between noble metal nanoparticles and semiconductor oxide due to the different work functions. The relatively high work function of noble metal particles (and even higher work functions of the oxidized noble metals) exceeds the work function of n-type semiconductor oxides. This leads to the transfer of electrons from the semiconductor oxide to the noble metal nanoparticles. It causes the band bending in the contact area of the noble metal nanoparticles and the semiconductor, which increases the thickness of the depleted layer near the surface of the semiconductor. If noble metal particle is partially oxidized (for example, PdO_x_), then in the presence of reducing gases, it can be reduced to the metallic form, which leads to a significant decrease of resistance of the noble metal/semiconductor junction. Palladium-modified zinc oxide ZnO/Pd in the form of nanofibers, nanowires, and nanorods has been studied as a sensitive material for detecting hydrogen (most commonly), as well as CO, hydrocarbons, and volatile organic compounds. The literature data on the sensor properties of ZnO/Pd materials are summarized in Table 1.

Traditionally, the effect of noble metal nanoparticles on the sensor properties of semiconductor oxides is considered on the example of measurements performed in dry air. The mechanism of formation of the sensor response of modified semiconductor oxides in the presence of humidity has rarely been discussed [14,15,16,17,18,19,20,21]. In this work, we determined the sensor properties of nanocrystalline fibers of unmodified and Pd-modified zinc oxide when detecting CO in dry (relative humidity at 25 °C RH_25_ = 0%) and humid (RH_25_ = 60%) air, and investigated the mechanism of sensor response formation in dry and humid atmosphere by in situ diffuse reflectance IR Fourier transform spectroscopy (DRIFTS).

## 2. Materials and Methods

### 2.1. Materials Synthesis

Nanocrystalline fibers of unmodified (ZnO) and Pd-modified (ZnO/Pd, 1 wt.%) zinc oxide were synthesized by electrospinning of the polymer solution followed by heat treatment to remove the polymer and crystallize the semiconductor material. Annealing conditions for polymer decomposition were determined by thermogravimetric analysis with mass spectral determination of gaseous products (TG-MS) using a NETZSCH STA 409 PC/PG instrument.

Zinc acetate Zn(CH_3_COO)_2_·2H_2_O and palladium acetylacetonate (Pd(acac)_2_, Pd(CH_3_COCHCOCH_3_)_2_) served as a source of zinc and palladium, respectively. When preparing a polymer solution, 200 mg of zinc acetate were dissolved in 10 mL of a mixed solvent consisting of 2-methoxyethanol and isopropanol (1:1). After complete dissolution of zinc acetate, 900 mg of polyvinylpyrrolidone (PVP) were added and the mixture was vigorously stirred for 5 h at 40 °C. Electrospinning proceeded at a solution feed rate of 1 mL/h at a distance of 125 mm and a voltage of 12 kV between the needle and the metal collector. The resulting polymer nanofibers were collected and annealed in air at 550 °C for 5 h (heating rate of 1 K/min). Palladium modified ZnO nanofibers were obtained by a similar method, after adding palladium precursor to the initial polymer solution.

### 2.2. Materials Characterization

The elemental composition of ZnO/Pd (1 wt.% Pd) was confirmed by X-ray fluorescence using the M1 Mistral micro-X-ray spectrometer (Bruker). The following parameters were used for the measurement: 50 kV tube voltage, 800 mA current, 1.5 mm diameter of the analyzed area, and 2 min signal accumulation time. Internal calibration of the device was used.

The phase composition and size of the crystal grains (*d_XRD_*) of the obtained samples were determined by X-ray diffraction (XRD) on the DRON-4M instrument (Cu K_α_ radiation, wavelength 1.54051 Å). The *d_XRD_* values for ZnO phase were estimated using the Scherrer formula.

The microstructure of the nanofibers was investigated using scanning electron microscopy (SEM) on Carl Zeiss NVision 40 microscope with an intra-lens detector at an accelerating voltage of 5 kV.

The distribution of palladium clusters in ZnO nanofibers was studied using high-resolution transmission electron microscopy (HRTEM), high angle annular dark field scanning transmission electron microscopy (HAADF-STEM), and energy-dispersive X-ray (EDX) mapping on a FEI Osiris microscope with a Super-X detector at 200 kV.

The surface composition of ZnO and ZnO/Pd nanofibers and the chemical state of the elements (Zn, O, Pd) were studied by X-ray photoelectron spectroscopy (XPS) using an Axis Ultra DLD spectrometer (Kratos, UK). The spectra were obtained using monochromatic Al K_α_ radiation under ultrahigh vacuum conditions (10^−8^ Torr), with an emission current of 10 mA. The charge shift was corrected using C 1s ground state peak with a binding energy of 285.0 eV. The spectra for the Zn 2p, O 1s and Pd 3d regions were recorded with a step of 0.1 eV. The spectra were fitted by mixed Gaussian-Lorentz functions.

Adsorbed species on the surface of ZnO and ZnO/Pd nanofibers pressed in KBr pellets were determined by Fourier-transform infrared spectroscopy (FTIR) technique using a Frontier spectrometer (Perkin Elmer). The spectra were recorded in the transmission mode in the 4000–400 cm^−1^ range with a step of 1 cm^−1^.

The sensor properties of ZnO and ZnO/Pd nanofibers were studied by in situ measurement of DC electrical conductivity under controlled temperature, humidity, and gas phase composition in an automatic set up with a flow chamber. To measure the electrical conductivity, ZnO and ZnO/Pd nanofibers were mixed with α-terpineol and drop-deposited onto alumina microhotplates 2 × 0.5 × 0.1 mm with top deposited Pt electrodes (the distance between the electrodes is 0.1 mm) and embedded Pt-meander serving as a heater. The microhotplate was mounted in a TO-8 package with four Pt wires (20 µm in diameter). The image and description of the chip can be found in [37]. The power consumption of the microhotplate is 240 mW at 300 °C. The obtained thick films were dried at room temperature for 24 h and then annealed at 450 °C for 24 h in purified dry air. The sensor resistance was measured at 1.3 V DC-voltage under controlled gas flow of 100 ± 0.1 mL/min at a temperature fixed in the range of 100–450 °C with the step of 50 °C. The gas sensor properties of ZnO and ZnO/Pd nanofibers toward carbon monoxide were studied in the concentration range of 5–15 ppm CO in dry (relative humidity at 25 °C RH_25_ = 0%) and humid (RH_25_ = 60%) air. The selected range of CO concentrations meets the World Health Organization (WHO) recommendations for indoor air quality (15 min—100 mg/m^3^ (86 ppm), 1 h—35 mg/m^3^ (30 ppm), 8 h—10 mg/m^3^ (9 ppm), 24 h—7 mg/m^3^ (6 ppm)) [38]. The CO concentration in air was set by electronic flow meters EL-FLOW (Bronkhorst). The humidity of CO containing gas mixtures and background air was pre-assigned and controlled by P-2 Cellkraft humidifier. The sensor signal *S* was calculated as

(1) $$S=\frac{R_{air}}{R_{gas}},$$

where *R_air_* is the sample’s resistance in background air, *R_gas_* is the sample’s resistance in CO containing gas mixture.

The interaction of ZnO and ZnO/Pd nanofibers with CO in dry and humid air was studied using the diffuse reflectance IR Fourier transform spectroscopy (DRIFTS) on a Perkin Elmer Frontier spectrometer with DiffusIR annex and HC900 flow chamber (Pike Technologies) with a heater and ZnSe window. Spectra were recorded in the range of 4000–1000 cm^−1^ with 1 cm^−1^ step and accumulation of 30 scans.

## 3. Results and Discussion

**Figure 1:**
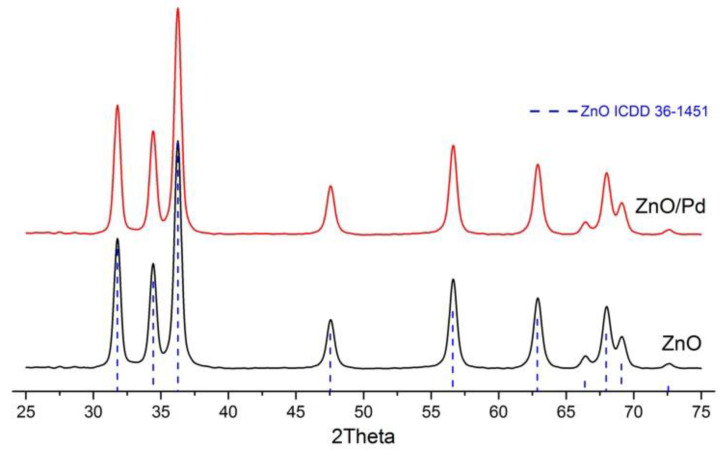
X-ray diffraction patterns of the ZnO and ZnO/Pd nanofibers. Dashed lines correspond to the reflection positions of ZnO (ICDD 36-1451).

According to XRD data, the obtained ZnO and ZnO/Pd nanofibers contained only one crystalline phase ZnO wurtzite (Figure 1). The crystallite size *d_XRD_* of ZnO phase was 15 ± 1 nm. Adding the palladium precursor to the polymer solution for electrospinning did not influence the crystallite size of the formed zinc oxide phase (*d_XRD_* = 13 ± 1 nm).

**Figure 2:**
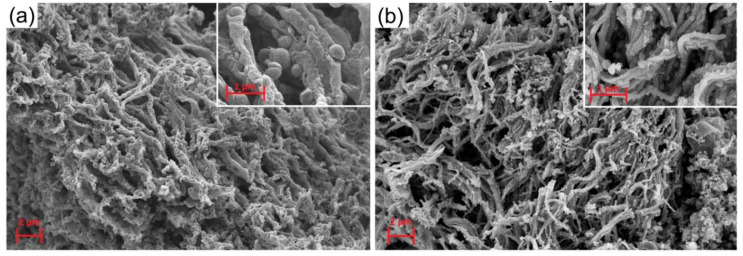
SEM images of ZnO (**a**) and ZnO/Pd (**b**) nanofibers.

The morphology of the ZnO and ZnO/Pd nanofibers is shown in Figure 2. Pure ZnO fibers with a diameter of 500–1000 nm form a three-dimensional spongy structure (Figure 2a). The introduction of Pd(acac)_2_ during synthesis lead to the formation of thinner ZnO fibers (150–200 nm in diameter), which also form a spongy structure (Figure 2b), but with a lower density compared to unmodified ZnO.

**Figure 3:**
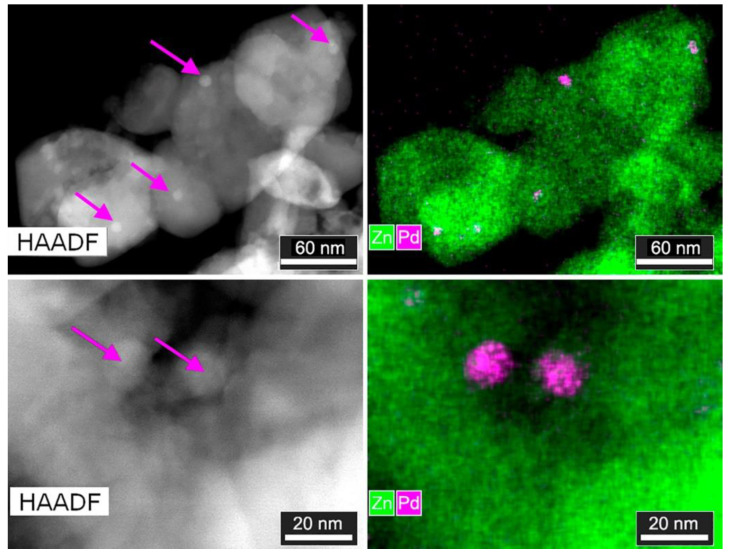
High angle annular dark field scanning transmission electron microscopy (HAADF-STEM) images and STEM-energy-dispersive X-ray (EDX) maps of ZnO/Pd sample at different magnifications. At HAADF-STEM image Pd nanoparticles are indicated with arrows.

According to HAADF-STEM and STEM-EDX mapping (Figure 3), the ZnO/Pd nanofibers are composed of the agglomerated crystalline ZnO particles with various shapes and sizes. The partial destruction of the nanofiber’s microstructure could be caused by the method of sample preparation for TEM analysis. The Pd clusters on the surface of ZnO grains can be seen directly on HAADF-STEM images because Z_Pd_ = 46 is higher than Z_Zn_ = 30. The particles have spherical shape with the diameter of 5–14 nm. Since Pd clusters are located on ZnO grains, it was not possible to determine whether they are metallic or oxide particles.

**Figure 4:**
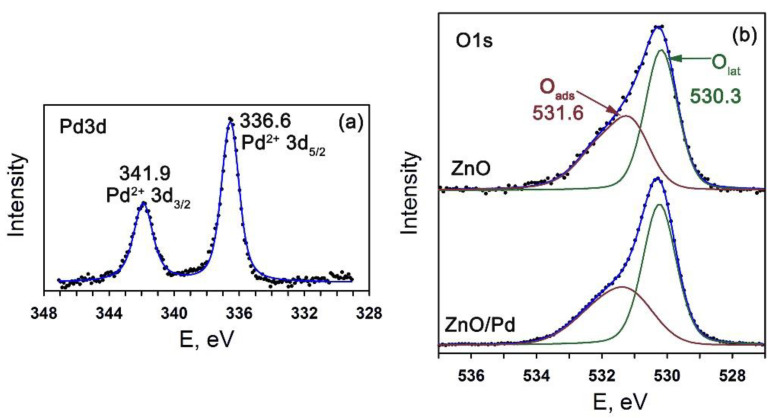
(**a**) Pd3d XP-spectrum of ZnO/Pd nanofibers. (**b**) O1s XP-spectra of the ZnO and ZnO/Pd nanofibers.

The Pd3d X-ray photoelectron spectrum of nanocomposites ZnO/Pd can be fitted by one doublet component (Figure 4a). Comparing the estimated Pd 3d_5/2_ and Pd 3d_3/2_ binding energies with reference data [39], one can conclude that palladium was in the Pd^2+^ oxidation state in ZnO/Pd nanofibers. The XP spectra of ZnO and ZnO/Pd in the O1s region are compared in Figure 4b. In both cases the O1s spectrum can be described by two components with maxima at 530.3 eV (O_lat_) and 531.6 eV (O_ads_). The O_lat_ component corresponds to the oxygen anions of the ZnO crystal structure, and the appearance of the O_ads_ component is due to various oxygen-containing species on ZnO surface, namely chemisorbed oxygen and different OH-groups. The area ratio of the components O_ads_/O_lat_ = 0.90 for pure ZnO. The introduction of palladium results in the decrease in the O_ads_/O_lat_ ratio down to 0.74 that indicates lowering the concentration of oxygen-containing species on the ZnO/Pd surface.

**Figure 5:**
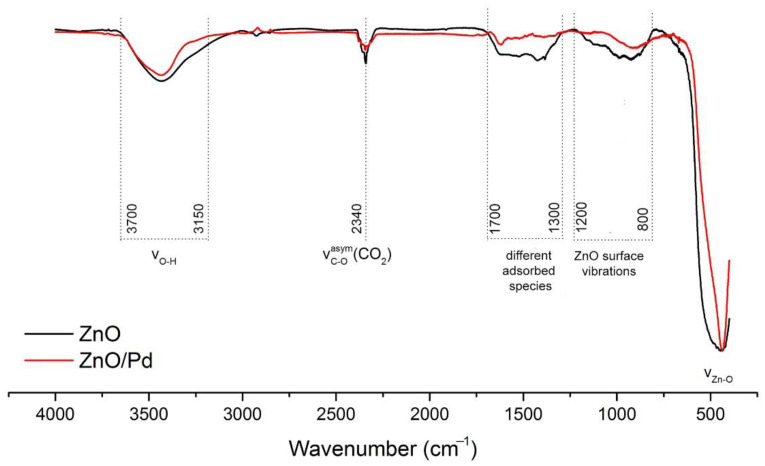
FTIR transmission spectra of ZnO and ZnO/Pd nanofibers.

In the FTIR transmission spectra (Figure 5), the bands of lattice Zn–O (420–460 cm^−1^) and surface Zn–O (800–1200 cm^−1^) vibrations [40] and/or phonon mode vibrations of ZnO (700–1100 cm^−1^) [41] were observed. The bands at 1300–1700 cm^−1^ could be caused by bending vibrations of adsorbed molecular water (1630 cm^−1^) and surface Zn–OH groups (1320, 1395, 1410, 1560, 1605, and 1640 cm^−1^; [42]) as well as carbonate (1350 and 1530 cm^−1^; [43]), hydrocarbonate (1635 cm^−1^; [44]) or adsorbed CO_2_ (1370 cm^−1^; [44]; 1360–1450 cm^−1^, 1540–1650 cm^−1^; [43]). The band at 2340 cm^−1^ corresponds to the asymmetric stretching vibrations of adsorbed CO_2_. The wide band of stretching O–H vibrations at 3150–3700 cm^−1^ indicates the presence of various hydrated surface species including separate OH-groups (3500–3700 cm^−1^; [43,45]), rooted OH...OH groups (3400 cm^−1^ [45]), and OH-groups associated with zinc vacancies (3220–3230 cm^−1^; [43]). The introduction of palladium into the ZnO nanofibers lead to the decrease in the relative intensities of hydroxyl related bands mainly at 3300–3100 cm^−1^ and 1600–1300 cm^−1^. This is consistent with XPS results. Thus, it can be assumed that the decrease in the concentration of oxygen-containing particles on the surface of ZnO/Pd in comparison with ZnO nanofibers is due to a decrease in the concentration of surface hydroxyl groups associated with zinc cations or their vacancies, and not a decrease in the amount of chemisorbed oxygen.

**Figure 6:**
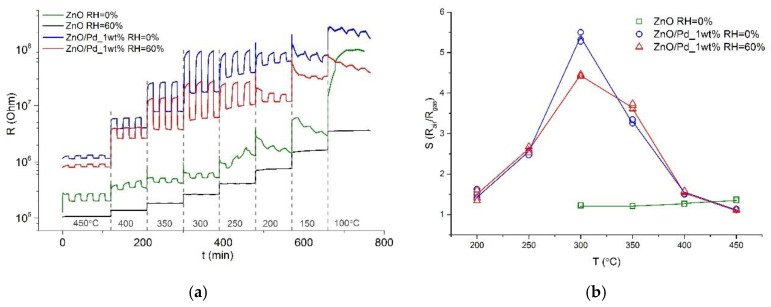
Transient sensor response (**a**) and temperature dependence of sensor signal (**b**) of ZnO and ZnO/Pd nanofibers to 5 ppm CO in dry (RH_25_ = 0%) and humid (RH_25_ = 60%).

The sensor properties of ZnO and ZnO/Pd nanofibers were investigated to carbon monoxide (5–15 ppm) in dry (RH_25_ = 0%) and humid (RH_25_ = 60%) conditions. Figure 6a shows the resistance change of ZnO and ZnO/Pd nanofibers with a cyclic variation of gas phase composition (air—5 ppm CO in air) in the temperature range 100–450 °C. Modification by palladium increased the resistance of ZnO nanofibers by an order of magnitude. This is due to the formation of a potential barrier at the ZnO/PdO_x_ interface due to the fact that the electron work function for PdO_x_ (6.04 eV) is significantly greater than the corresponding value for ZnO (4.6 eV). For the ZnO nanofibers, the maximum signal in dry air was observed at T = 450 °C. In the temperature range T < 300 °C ZnO nanofibers demonstrated very slow conductivity change that makes meaningless the calculation of the sensor response. The modification of ZnO by palladium lead to a significant increase of the sensitivity to CO in the temperature range T = 250–350 °C (Figure 6b). Maximum response of ZnO/Pd nanofibers to CO was observed at T = 300 °C. An increase in air humidity leads to the complete disappearance of the sensitivity of ZnO nanofibers to carbon monoxide in the whole temperature range under study. At the same time, the sensor response of ZnO/Pd nanofibers changed slightly with increasing air humidity up to RH_25_ = 60% (Figure 6).

**Figure 7:**
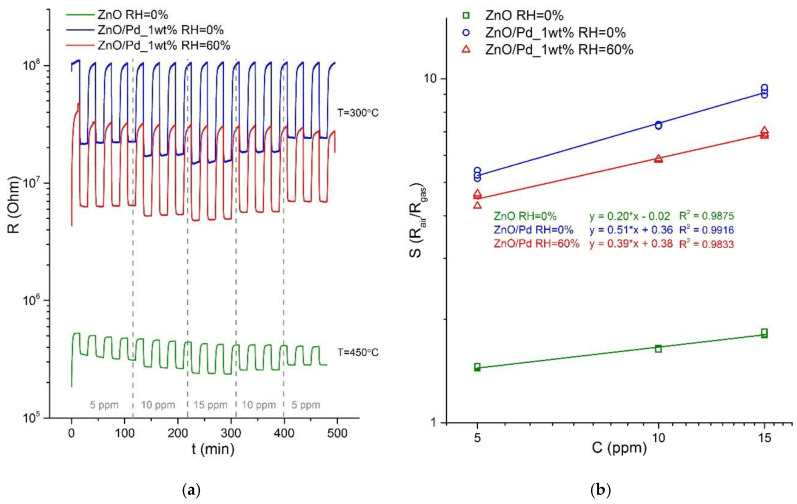
Transient sensor response (**a**) and calibration curves (**b**) for CO detection for ZnO and ZnO/Pd nanofibers at T = 450 °C and T = 300 °C, respectively, in dry (RH_25_ = 0%) and humid (RH_25_ = 60%) conditions.

The dependences of the sensor response of ZnO and ZnO/Pd nanofibers on the carbon monoxide concentration (measured at 450 °C for ZnO nanofibers and at 300 °C for ZnO/Pd nanofibers, Figure 7a) were linear in the double logarithmic scale (Figure 7b) corresponding to the power law $S\sim C_{CO}^{n}$ [46]. The minimum detectable CO concentrations were estimated from calibration curves presented in Figure 7b using the ratio $S_{min}=\frac{R_{av}}{R_{av}-3\sigma }$ as minimum measurable response (*R_av_* is the average value of *R_air_*; *σ* is the standard deviation of *R_air_*) [47]. In dry air, the minimum detectable CO concentrations were estimated as 2.7 ppm and 0.15 ppm for the sensors based on ZnO and ZnO/Pd nanofibers, respectively. When humidity increased to RH_25_ = 60%, this value for ZnO/Pd nanofibers increased up to 0.51 ppm CO. Note that this is true only if the dependence of the sensor signal on the CO concentration in this range remains linear in the double logarithmic scale.

**Figure 8:**
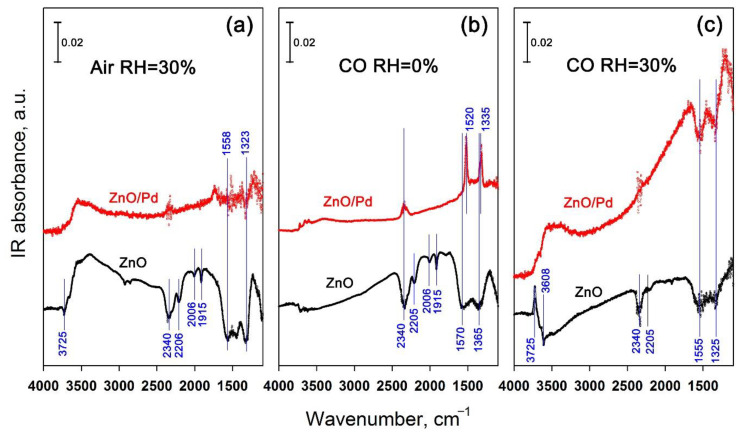
Diffuse reflectance IR Fourier transform spectroscopy (DRIFTS) of ZnO and ZnO/Pd nanofibers in humid air (**a**), in the presence of 500 ppm CO in dry air (RH_25_ = 0%) (**b**), in the presence of 500 ppm CO in humid air (RH_25_ = 60%) (**c**).

The interaction of ZnO and ZnO/Pd nanofibers with CO in dry and humid air was investigated by the DRIFTS method (Figure 8). The experiments were performed at temperatures corresponding to the maximum sensor signal in dry air, i.e., at T = 450 °C and T = 300 °C for ZnO and ZnO/Pd nanofibers, respectively.

Figure 8a shows the DRIFT spectra in wet air of ZnO and ZnO/Pd nanofibers. Spectra in dry air at T = 450 °C and T = 300 °C, respectively, were used as the baselines. The spectrum of ZnO nanofibers is complex. A positive broad band in the region of 3700–3000 cm^−1^ implies an increasing concentration of OH groups formed on the ZnO surface in humid air. An intense negative band at 2340 cm^−1^ indicates desorption of CO_2_ molecules formed and trapped on the surface of ZnO nanofibers during the decomposition of polymer precursor. The authors [48] have shown that such CO_2_ molecules are stable on the surface of defective ZnO powders even at elevated temperatures. Negative bands at 2205, 2006, and 1915 cm^−1^ indicate desorption of chemisorbed oxygen related to nitrogen impurities in zinc oxide produced during PVP decomposition [49]. Thus, it can be assumed that at 450 °C in a humid atmosphere, O_2_ adsorbed on the surface of ZnO nanofibers is replaced by OH groups.

The nature of the two negative bands in the 1600–1300 cm^−1^ region is not entirely clear [50,51]. However, it is worth noting that the positive absorption intensity in this region increases with the increase in the partial pressure of oxygen in the surrounding atmosphere [52], while the negative intensity is observed in the presence of reducing gases CO and H_2_ [50] and increases due to continuous heating in high vacuum at T = 370 °C [52]. Thus, the nature of these bands is related to defects in the crystal structure of ZnO [51]. It can also be assumed that these bands are associated with the oxygen of the ZnO crystal lattice, since the adsorption of water on the surface of zinc oxide proceeds by a dissociative mechanism, which involves the interaction between water vapor (H_2_O_gas_) and adsorbed (O^α-^_ads_) or lattice (O_lat_) oxygen [53,54]

(2) $$H_{2}O_{\mathrm{gas}}+2{\mathrm{Zn}}_{\mathrm{lat}}+O_{\mathrm{ads}}^{-\alpha }\to 2\left({\mathrm{Zn}}_{\mathrm{lat}}\mathrm{OH}\right)+{\alpha e}^{-}$$

(3) $$H_{2}O_{\mathrm{gas}}+\left(O_{\mathrm{lat}}-{\mathrm{Zn}}_{\mathrm{lat}}-O_{\mathrm{lat}}\right)\to \left(O_{\mathrm{lat}}-{\mathrm{Zn}}_{\mathrm{lat}}\mathrm{OH}-O_{\mathrm{lat}}H\right)$$

(4) $$H_{2}O_{\mathrm{gas}}+2{\mathrm{Zn}}_{\mathrm{lat}}+O_{\mathrm{lat}}\to 2\left({\mathrm{Zn}}_{\mathrm{lat}}\mathrm{OH}\right)+V_{O}^{2+}+2e^{-}$$

The DRIFT spectrum of ZnO/Pd nanofibers obtained in humid air at T = 300 °C showed only a wide positive band in the range 3700–3000 cm^−1^, indicating an increased concentration of OH groups, and weak negative bands in the 1600–1300 cm^−1^ region indicating a change in the electronic state of lattice oxygen in subsurface layers of ZnO grains. In general, the presence of Pd clusters on the surface of ZnO nanofibers lead to decreased reactivity in the interaction with water vapor.

The DRIFT spectrum of ZnO nanofibers interacting with 500 ppm CO in dry air (Figure 8b) is identical to the spectrum obtained in wet air in the absence of carbon monoxide (Figure 8a), except for the band corresponding to the increased concentration of OH groups. This indicates that at 450 °C, CO oxidation on the ZnO surface occurs both with the participation of chemisorbed oxygen (Reaction (5)) and by the Mars–van Krevelen mechanism with the participation of lattice oxygen (Reaction (6)). The observed modulation of the spectrum baseline is due to increasing charge density in the semiconductor oxide [52].

(5) $${\mathrm{CO}}_{\mathrm{gas}}+O_{\mathrm{ads}}^{-\alpha }\to {\mathrm{CO}}_{2\;\mathrm{gas}}+{\alpha e}^{-}$$

(6) $${\mathrm{CO}}_{\mathrm{gas}}+O_{\mathrm{lat}}\to {\mathrm{CO}}_{2\;\mathrm{gas}}+V_{O}^{2+}+2e^{-}$$

The DRIFT spectrum of ZnO/Pd nanofibers obtained in the presence of 500 ppm CO in dry air at T = 300 °C differs significantly (Figure 8b). The spectrum contains a positive band at 2340 cm^−1^, corresponding to adsorbed CO_2_, and sharp intense positive bands at 1520 and 1355 cm^−1^, corresponding to stretching vibrations of bidentate carbonate groups [55] while negative bands in the 1600–1300 cm^−1^ region do not appear. Baseline modulation is less pronounced than in the case of ZnO. From these data, it can be assumed that CO oxidation on the surface of ZnO/Pd nanofibers occurs with the participation of PdO_x_ clusters.

(7) $${\mathrm{CO}}_{\mathrm{gas}}+{\mathrm{PdO}}_{x}\to {\mathrm{CO}}_{2\;\mathrm{gas}}+\mathrm{Pd},$$

and carbon dioxide formed by the Reaction (7) is adsorbed on the surface of ZnO on lattice oxygen anions O_lat_ [56].

(8) $${\mathrm{CO}}_{2\;\mathrm{gas}}+O_{\mathrm{lat}}\to {\mathrm{CO}}_{3\;\mathrm{ads}}.$$

The effect of palladium clusters on the interaction of ZnO with carbon monoxide corresponds to the mechanism of electronic sensitization. The work function of the reduced palladium surface (4.8 eV) is less than in that of PdO_x_ (6.04 eV). The work function of ZnO is about 4.6 eV [57]. Thus, as a result of reduction of PdO_x_ clusters by the Reaction (7), the barrier at the Pd/ZnO contact disappears, which leads to a resistance decrease registered as the sensor response.

The DRIFT spectra of ZnO and ZnO/Pd nanofibers in the presence of 500 ppm CO in humid air are shown in Figure 8c. The corresponding spectra from Figure 8a were used as the baseline. The following features can be noted for the spectrum of ZnO nanofibers.

Differences in the DRIFT spectra of ZnO nanofibers obtained in the presence of CO in a dry and humid atmosphere can be summarized as follows. (i) In humid air, there is a wide negative band in the range 3700–3300 cm^−1^ with a narrow minimum at 3608 cm^−1^, corresponding to the stretching vibrations of OH groups formed on polar O-ZnO ($000\bar{1})$ surface via dissociation of water on oxygen vacancy sites [10]. At the same time, a sharp maximum appeared at 3725 cm^−1^, indicating an increasing concentration of OH groups on the mixed-terminated ZnO $\left(00\bar{1}0\right)$ surface [10]. Thus, in the presence of CO in humid air, the hydroxyl layer on the surface of the crystalline grains of zinc oxide is rearranged. (ii) Negative bands at 2205, 2006, and 1915 cm^−1^, corresponding to desorption of chemisorbed oxygen related to nitrogen impurities in zinc oxide, disappeared almost completely. This means that in the atmosphere of humid air there is a complete replacement of chemosorbed oxygen with hydroxyl groups and the introduction of CO does not further affect this characteristic of ZnO surface. Thus, oxidation of carbon monoxide with chemisorbed oxygen (Reaction (5)) cannot contribute to the sensor response. (iii) There is a significant decrease in the intensity of negative bands in the region of 1600–1300 cm^−1^ associated with extraction of the oxygen from the ZnO crystal lattice (Reaction (6)). This indicates that the oxidation of CO on the ZnO surface by the Mars–van Krevelen mechanism is suppressed. Therefore, carbon monoxide and ZnO surface do not interact by Reactions (5) and (6) accounting for the almost complete loss of sensor sensitivity of ZnO nanofibers when detecting CO in humid air.

The DRIFT spectra of ZnO/Pd nanofibers in the presence of CO in dry and humid air differ dramatically. First of all, it should be noted that increasing humidity leads to the absence of sharp positive signals from surface carbonates. Instead, wide negative bands appear in the area of 1600–1300 cm^−1^, which are typical for ZnO in a reducing atmosphere. In addition, there is an intense positive band in the range of 3700–3300 cm^−1^, which corresponds to an increase of the concentration of surface OH groups. Finally, we should note a powerful modulation of the baseline due to an increase of the concentration of free charge carriers in ZnO.

The retention of the value of the sensor response of ZnO/Pd nanofibers in humid air, in contrast to the case of unmodified ZnO, indicates that the effect of electronic sensitization by PdO_x_ clusters persists, and the Reaction (7) is the main process that causes the change of electrophysical properties of ZnO/Pd in the presence of CO. The authors of [58] investigated the effects of adsorbed H_2_O on the CO oxidation on PdO(101). It was found that water molecules inhibit the adsorption of CO, but also provide a facile pathway for CO oxidation on PdO(101). The oxidation of CO in co-adsorbed CO + H_2_O layers on PdO(101) produces CO_2_ at a temperature of ~50 K lower than CO oxidation on pristine PdO(101). The absence of surface carbonates on ZnO/Pd in humid air may be due to the ratio of enthalpy of H_2_O and CO_2_ adsorption on the ZnO surface [59]. On ZnO treated in vacuum at 450 °C the values of integral enthalpy of H_2_O and CO_2_ adsorption are very close: −96.8 ± 2.5 and −96.6 ± 2.5 kJ/mol, respectively. Adsorption of CO_2_ on a partially hydrated ZnO surface results in integral enthalpy of adsorption of only −47.4 ± 1.2 kJ/mol and in lower coverage of 0.9 CO_2_/nm^2^ instead of 2.6 CO_2_/nm_2_ on vacuum treated sample [59]. Thus, it can be assumed that CO_2_ formed by the Reaction (7) is not adsorbed on the surface of ZnO under high humidity conditions.

The appearance of negative bands in the region of 1600–1300 cm^−1^ and a strong baseline modulation of the DRIFT spectrum due to an increase of the concentration of charge carriers indicate the involvement of an additional mechanism for CO oxidation on the ZnO surface. Similar to SnO_2_/Pd [14,60], it can be assumed that there is a direct oxidation of carbon monoxide by OH-groups formed on ZnO surface during dissociative adsorption of water (Reaction (9)):

(9) $${\mathrm{CO}}_{\mathrm{gas}}+\mathrm{OH}\to {\mathrm{CO}}_{2\;\mathrm{gas}}+H^{+}+e^{-}$$

This likely becomes possible due to the weakening of the С–О bond during CO adsorption on the surface of reduced Pd^0^ clusters [61].

## 4. Conclusions

In situ DRIFTS investigation of the interaction of ZnO and ZnO/Pd nanofibers with carbon monoxide in dry and humid air allowed us to make the following conclusions. The formation of the sensor response of unmodified ZnO when detecting CO in dry air is due to CO oxidation by chemisorbed and lattice oxygen (Mars–van Krevelen mechanism). Under high humidity (RH_25_ = 60%) conditions, both of these mechanisms are suppressed resulting in a complete loss of ZnO sensitivity to CO in the temperature range of 100–450 °C. In the case of ZnO/Pd, the formation of high sensor response in dry and humid air is due to the electronic sensitization effect, namely, the decrease in the Schottky barrier at ZnO/Pd interface due to the reduction of PdO_x_ clusters by carbon monoxide.

## Figures and Tables

**Table 1:** Summary of ZnO/Pd materials for gas sensors.

Material Type	Sensor Signal ^(1)^	Gas (Concentration)	Measurement Temperature, °C	Ref.
Schottky contact	3.25	H_2_ (50 ppm)	150	[22]
	1.41	CH_4_ (50 ppm)		
Schottky contact	1.45	NO_2_ (50 ppm)	RT ^(2)^	[23]
Nanorods	4.6	H_2_ (500 ppm)	350	[24]
Nanorods	2.04	H_2_ (500 ppm)	RT	[25]
Nanorods	15.02	H_2_ (360 ppm)	RT	[26]
Nanorods	1106	H_2_ (500 ppm)	260	[27]
Nanorods	5.5	CO (100 ppm)	260	[28]
Nanowires	12.2	H_2_ (4000 ppm)	RT	[29]
Nanowires	1.02	CO (0.1 ppm)	RT	[30]
Nanowires	87.17	H_2_ (100 ppm)	350	[31]
Nanowires	13.5	NO_2_ (1 ppm)	100	[30]
Nanowires	13100	H_2_ (100 ppm)	RT	[32]
Nanowires	4.3	H_2_ (10 ppm)	200	[33]
Nanowires	5.25	Ethanol (200 ppm)	325	[34]
	3.75	Acetone (200 ppm)		
	3	Methanol (200 ppm)		
Nanocomposite	37	H_2_ (1 vol.%)	250	[35]
			500	
			550	
	15	C_3_H_8_ (1 vol.%)		
	13	CO (1 vol.%)		
Nanocomposite	8	CO (1000 ppm)	400	[36]

^(1)^ Recalculated for all cases as *S* = *R_air_*/*R_gas_*. ^(2)^ Room temperature.
